# Identification of a novel signature based on macrophage-related marker genes to predict prognosis and immunotherapeutic effects in hepatocellular carcinoma

**DOI:** 10.3389/fonc.2023.1176572

**Published:** 2023-05-25

**Authors:** Yuanshuai Su, Chen Xue, Xinyu Gu, Wankun Wang, Yu Sun, Renfang Zhang, Lanjuan Li

**Affiliations:** ^1^ State Key Laboratory for Diagnosis and Treatment of Infectious Diseases, National Clinical Research Center for Infectious Diseases, National Medical Center for Infectious Diseases, Collaborative Innovation Center for Diagnosis and Treatment of Infectious Diseases, The First Affiliated Hospital, Zhejiang University School of Medicine, Hangzhou, China; ^2^ Department of Surgical Oncology, The First Affiliated Hospital, School of Medicine, Zhejiang University, Hangzhou, Zhejiang, China

**Keywords:** HCC, ScRNA-seq, macrophage, prognosis, immunotherapy

## Abstract

**Background:**

Tumor-related macrophages (TAMs) have emerged as an essential part of the immune regulatory network in hepatocellular carcinoma (HCC). Constructing a TAM-related signature is significant for evaluating prognosis and immunotherapeutic response of HCC patients.

**Methods:**

Informative single-cell RNA sequencing (scRNA-seq) dataset was obtained from the Gene Expression Omnibus (GEO) database, and diverse cell subpopulations were identified by clustering dimension reduction. Moreover, we determined molecular subtypes with the best clustering efficacy by calculating the cumulative distribution function (CDF). The ESTIMATE method, CIBERSORT (cell-type identification by estimating relative subsets of RNA transcripts) algorithm and publicly available tumor immune dysfunction and exclusion (TIDE) tools were used to characterize the immune landscape and tumor immune escape status. A TAM-related gene risk model was constructed through Cox regression and verified in multiple datasets and dimensions. We also performed functional enrichment analysis to detect potential signaling pathways related to TAM marker genes.

**Results:**

In total, 10 subpopulations and 165 TAM-related marker genes were obtained from the scRNA-seq dataset (GSE149614). After clustering 3 molecular subtypes based on TAM-related marker genes, we found significantly different prognostic survival and immune signatures among the three subtypes. Subsequently, a 9-gene predictive signature (TPP1, FTL, CXCL8, CD68, ATP6V1F, CSTB, YBX1, LGALS3, and APLP2) was identified as an independent prognostic factor for HCC patients. Those patients with high RiskScore had a lower survival rate and benefited less from immunotherapy than those with low RiskScore. Moreover, more samples of the Cluster C subtype were enriched in the high-risk group, with higher tumor immune escape incidence.

**Conclusions:**

We constructed a TAM-related signature with excellent efficacy for predicting prognostic survival and immunotherapeutic responses in HCC patients.

## 1 Introduction

Primary liver cancer, a type of highly malignant tumor, has been the major cause of tumor-related mortality (1, 2). According to global statistics, with 905,677 newly diagnosed cases and 9,30,180 death cases in 2020, liver cancer has caused a heavy burden on the global health system. Hepatocellular carcinoma (HCC), accounting for approximately 80% of all cases, is the most common histopathological subtype of primary liver cancer and is characterized by high aggressiveness, low treatment response and poor outcome (3, 4). Unfortunately, most patients with HCC are at terminal stages due to the lack of available diagnostic and therapeutic measures, leading to a disappointingly low survival rate (1-year survival rate <20%) (5). Despite considerable advances in hepatocarcinogenesis in recent decades, further exploration and construction of novel strategies to monitor and apply intervention in patients with HCC are needed.

The tumor immune microenvironment (TIME) is mainly composed of cancer cells, inflammatory cells, immune cells and the extracellular matrix (6, 7). The complex and dynamic interactions of various immune cells and active factors involved in immune regulation play an essential role in oncogenesis, metastasis and the treatment response (8, 9). Macrophages are important immune cells that participate in various immune activation processes, exerting important functions of phagocyte fragments, mediating inflammatory reactions and regulating tissue repair and regeneration (10, 11). Tumor-associated macrophages (TAMs), which are abundantly infiltrating immune cells in the TIME, are crucial factors in tumor-associated inflammation and modulate the development of cancers by secreting various cytokines and influencing other immune cells (10). Among the disparate functional phenotypes of TAMs after polarization, the M1 type (classical activated macrophages) and the M2 type (induced by IL-4 and IL-13) are most concerned (12). As tumors progress, M2-like TAMs contribute to cancer metastasis by facilitating the epithelial–mesenchymal transition (EMT) and angiogenesis (13, 14). More importantly, TAMs induce dysfunction of natural killer (NK) cells and restrain the effector T-cell response by attracting immunosuppressive cells such as T regulatory cells (Tregs) to the TIME, thereby decreasing antitumor immune effects and accelerating oncogenesis (15, 16). It is imperative to explore the molecular features of TAMs in HCC and construct a TAM-related predictive signature.

Traditional RNA-sequencing technology (bulk RNA-seq) is based on heterogeneous tissues or cell populations and reflects average transcription profiles at an integrated level (17). However, extensive heterogeneity exists among cell subpopulations, which is of great significance for driving the phenotypes of cancers. Remarkably, single-cell RNA sequencing (scRNA-seq) can reveal the expression signature of all genes at the single-cell level, with a more intuitive view of intratumor heterogeneity and individual cellular subpopulations (18–20), greatly facilitating relevant research and application. Moreover, informative datasets based on scRNA-seq are crucial for studying the functional characteristics of distinct cell subpopulations and the cell-interactive networks in tumors.

Herein, we comprehensively analyze scRNA-seq and bulk RNA data with informative data on clinical phenotypes. After clustering and dimensionality reduction, we annotated various cell subtypes through specifically expressed marker genes. Moreover, based on TAM-related marker genes, we defined three molecular subtypes and constructed a risk model to predict prognosis, immune landscape and biological activities in HCC. The predictive capability of the gene signature was further evaluated through multidimensional and multidataset validation.

## 2 Materials and methods

### 2.1 Data collection and preprocessing

We downloaded the scRNA-seq dataset GSE149614, which contains informative data for 21 HCC samples, from the Gene Expression Omnibus (GEO) database (https://www.ncbi.nlm.nih.gov/gds/) (21). Next, we filtered the scRNA-seq data according to the standard that each gene is expressed in more than 3 cells and that the expressed genes number less than 6000 and more than 100 in each cell. The PercentageFeatureSet function was utilized to calculate the ratio between rRNA and mitochondria to assure that the content of mitochondria was less than 10%. Additionally, the UMI number of each cell was at least 100 and less than 50,000. Finally, 64,424 cells were obtained from the original data.

For validation, gene expression spectra with informative data on clinical phenotypes were retrieved from the liver hepatocellular carcinoma (LIHC) cohort in The Cancer Genome Atlas (TCGA) (https://portal.gdc.cancer.gov/) and HCCDB (http://lifeome.net/database/hccdb) databases (22, 23). Next, samples without follow-up information on prognosis were removed, and we used the mean value of expression data with multiple gene symbols. After screening, 365 samples from the LIHC cohort from TCGA and 389 samples from the LIHC cohort from HCCDB were finally incorporated in our study.

### 2.2 Clustering dimensionality reduction of single-cell sequencing data

First, we performed log-normalization to standardize the scRNA-seq data and found hypervariable genes by employing FindVariableFeatures functions, which identify variable characteristics through variance stabilization transformation. Then, we removed batches of samples using the CCA method through the FindIntegrationAnchors function and integrated 21 samples using the IntegrateData method. After scaling all genes through the ScaleData function, PCA dimensionality reduction was performed to find anchor points. We obtained 16 cell subpopulations clustered by the FindNeighbors and FindClusters functions (Resolution=0.2) (24). UMAP dimensionality reduction was performed on all cells with the RunUMAP function, which maps available high-dimensional data samples into low-dimensional space and achieves the dimensionality reduction effect. Finally, all cell subsets were annotated using canonical marker genes which were screened by using the FindAllMarkers function (logfc=0.5, Minpct=0.5) (25).

### 2.3 ConsensusClusterPlus and cumulative distribution function (CDF)

Marker genes of TAMs were uniformly clustered through the ConsensusClusterPlus R package. In addition, the pam algorithm and “pearson” were used to assess the measured distance. Next, we carried out 500 bootstraps, of which each single process included 80% of the patients in the training set. The 365 HCC samples in the LIHC cohort from TCGA were clustered by the ConsensusClusterPlus R package, and the optimal clustering classification was determined by calculating the consistency matrix and consistency CDF. By monitoring the distribution of the CDF delta area curve, we searched for relatively stable clustering results.

### 2.4 Cell-type identification by estimating relative subsets of RNA transcripts (CIBERSORT)

CIBERSORT is an effective assessment method for characterizing the cell subpopulation composition in multicomponent tissues based on the input matrix of transcript profiles, which is useful for exploring novel cell biomarkers (26). For this study, we calculated the scores of 22 immune cells using the CIBERSORT algorithm. The Kruskal test was performed to determine correlations between immune infiltration and molecular subtype and risk score.

### 2.5 Evaluation of tumor immune dysfunction and exclusion (TIDE)

The publicly available tool TIDE (http://tide.dfci.harvard.edu/algorithm) was employed to predict potential clinical treatment effects in different HCC molecular subtypes and risk groups. By using the TIDE framework, one can predict accurately (27, 28) immunotherapeutic response or resistance in patients with cancer. High predictive TIDE scores indicate a higher incidence of tumor immune escape, suggesting that patients will benefit less from immunotherapy than those with low TIDE scores.

### 2.6 Identification and validation of the risk model

To further screen TAM-related marker genes associated with prognosis, we conducted univariate Cox regression analysis with the Survival R package in the TCGA-LIHC dataset. LASSO (Least absolute shrinkage and selection operator) regression analysis is a biased computation that retains the advantages of subset contraction. Unique variable selection characteristics can better solve the complex multicollinearity problem during data processing (29), which is commonly used to screen survival-related genes and construct prognostic models. To further compress the number of key genes in the risk model, we performed LASSO analysis using the Glmnet R package and determined the lambda value when the model was optimal. Finally, we calculated the coefficients of these target genes through multivariate Cox regression analysis.

Next, to validate the stability of the risk model, a calculation was performed separately for each patient in the training datasets (TCGA-LIHC, HCCDB-LIHC and GSE76427) using the following formula: RiskScore = Σ coefficient_mRNAn_ * expression level _mRNAn_. In addition, we carried out receiver operating characteristic (ROC) analysis with the timeROC R package and evaluated the prognostic classification performance of the risk model for 1-, 3- and 5-year survival prediction.

### 2.7 Functional enrichment analysis

We screened differentially expressed genes (DEGs) among diverse subtypes through the Limma R package. Then, Kyoto Encyclopedia of Genes and Genomes (KEGG) functional pathway enrichment analysis was conducted by the WebGestaltR R package (screened by FDR<0.001) (30). To further explore signaling pathways potentially regulated by the risk model, we downloaded HALLMARK- and KEGG-related gene datasets from the Gene Set Enrichment Analysis (GSEA) official website and performed GSEA for high- and low-risk groups through the ClusterProfiler and fgsea R package (31).

### 2.8 Cell culture and reverse-transcription quantitative PCR (RT−qPCR)

The human hepatoma cell lines Hep-G2 and Huh-7 and normal hepatic cell line LO-2 (obtained from the Chinese Academy of Sciences) were cultured for measuring relative expression levels of genes. Modified medium (Gibco, USA) was supplemented with 1% antibiotics (Sigma, USA) and 10% fetal bovine serum (Wisent, Canada). All cells were cultured in a humidified incubator (5% CO2; 37°C). We used TRIzol reagent (QIAGEN, Germany) to extract total RNA from cell lines and an ABI7500 fast PCR instrument to perform RT−qPCR. GAPDH was used for normalization. The primer sequence information is shown in Additional file 1: **Supplementary Table 1**.

### 2.9 Statistical analysis

The R program (v 4.0.3) was used for statistical analysis and informative visualization in our study. Single-cell analysis package Seurat-V3 was performed in the study. Unsupervised Cox regression analysis was performed to determine the predictive performance of the risk model. Classified and continuous variables between subgroups were compared by Wilcoxon and t tests, respectively. A P value < 0.05 was considered statistically significant.

## 3 Results

### 3.1 Dimensionality reduction for clustering cell subsets and functional enrichment

The statistics of the cell data before and after filtering are shown in the histogram in **Supplementary Figure 1A**. After PCA dimensionality reduction, we drew the anchor plot of the first 50 PCs (**Supplementary Figure 1B**) and then removed the batch of samples through CCA methods (**Supplementary Figures 1C, D**). A total of 21 samples were integrated together by using IntegrateData. We selected dim=30 for UMAP dimensionality reduction, and a total of 16 cell subpopulations were obtained. Then, we used the classic marker genes to annotate these cell subgroups, as shown in **Supplementary Figure 2**. Subgroups 2, 3, 5 and 12 were identified as T cells by specifically expressed genes, including CD2, CD3D, CD3E and CD3G. Subgroup 11 was identified as B cells by specifically expressed genes, including CD79A and MS4A1. Subgroup 9 included plasma cells specifically expressing CD79A and JSRP1. Subgroup 10 included FB cells specifically expressing the genes ACTA2, PDGFRB and NOTCH3. Subgroup 6 included endoepithelial cells specifically expressing the PECAM1 gene. Subgroup 0 included hepatoma cells specifically expressing GPC3, CD24 and MDK. Subgroups 1, 4, 13 and 14 were macrophages specifically expressing CD163 and CD68. Mast cells, proliferating cells and NK cells were also identified by marker genes.

**Figure 1:**
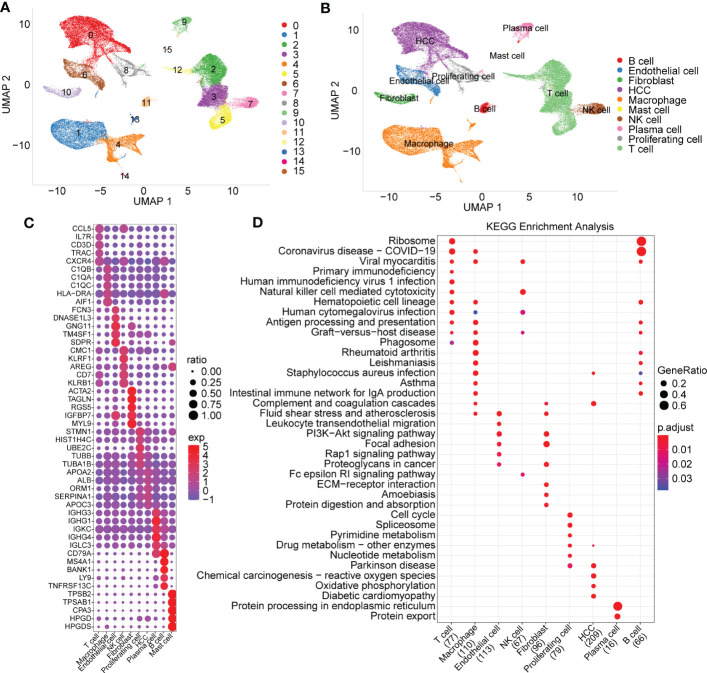
Cell subset distribution with functional annotation of marker genes. **(A)** UMAP map of the cell distribution of 16 cell subpopulations. **(B)** The cell subgroups identified by specially expressed marker genes. **(C)** Bubble diagram showing top5 marker genes in each cell subgroup (logfc=0.5, Minpct=0.5). **(D)** KEGG function enrichment analysis based on marker genes of each cell subgroup.

The UMAP map of the cell subpopulation distribution after clustering is shown in **Figure 1A**, and the annotated cell subgroups are shown in the form of UMAP (**Figure 1B**). We obtained a total of 10 cell subgroups, including B cells, endothelial cells, fibroblasts, HCC, macrophages, mast cells, NK cells, plasma cells, proliferating cells and T cells. Next, we screened marker genes of the 10 cell subpopulations by using the FindAllMarkers function (logfc=0.5, Minpct=0.5). The expression signatures of the first five prominent marker genes in each subpopulation are shown in **Figure 1C**. KEGG functional enrichment analysis results based on the marker genes of each cell population are provided in **Figure 1D**.

### 3.2 Construction of molecular subtypes based on TAM-related marker genes

**Figure 2:**
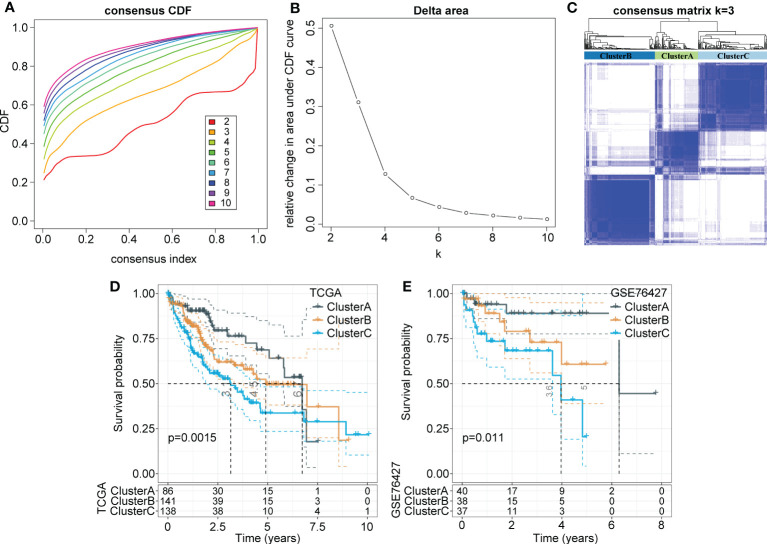
Identification of distinct molecular subtypes based on TAMs-related marker genes. **(A)** CDF curves of 365 samples in the LIHC cohort from TCGA database. **(B)** Delta area curve shows clustering results were relatively stable when Cluster number is selected as 3. The horizontal axis refers to the category number and the vertical axis refers to the relative change in the area under the CDF curve. **(C)** The sample clustering heatmap as consensus number=3. **(D, E)** Kaplan-Meier survival analysis of three subtypes in the LIHC cohort from TCGA. the independent LIHC dataset GSE76427.

Consistency clustering was carried out for the 165 marker genes of macrophages, and the best classification was determined by using the ConsensusClusterPlus R package. Then, we conducted consistency clustering on 365 HCC samples in the LIHC cohort from TCGA. The delta area curve of consensus clustering showed that the clustering results were relatively stable when the number selected was 3 (**Figures 2A, B**). Finally, we chose k=3 to define three molecular subtypes (**Figure 2C**). We further analyzed the prognostic signatures of these three subtypes and found significantly different prognostic survival of patients among molecular subtypes (**Figure 2D**). The prognosis of patients with HCC in Cluster C was the worst, followed by Cluster B; in contrast, the patients in Cluster A had a relatively good prognosis. For further verification, we used the same method to analyze the independent dataset GSE76427, with similar observations of prognostic outcomes in these three subtypes (**Figure 2E**).

### 3.3 Different clinical characteristics among molecular subtypes

**Figure 3:**
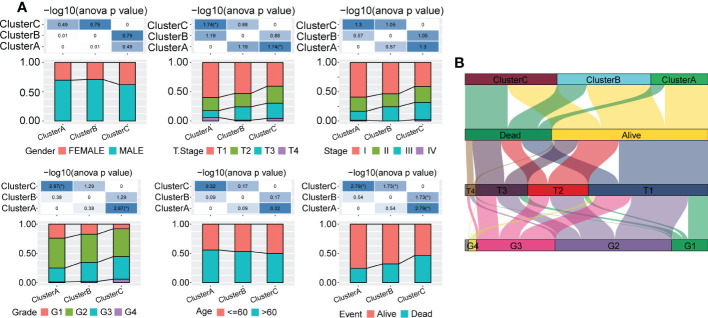
Distribution of multiple clinical phenotypes in the TCGA-LIHC cohort among three molecular subtypes. **(A)** There were significant differences of clinical features among patients in three Clusters. **(B)** Sangi diagram of distribution relationship between three molecular subtypes and clinical features including tumor stage, grade and survival status of patients.

Next, we explored the distribution characteristics of multiple clinical phenotypes in three molecular subtypes (chi-square test) and found significant differences in clinical features, including the tumor stage, grade and survival status, among the three subtypes (**Figure 3A**). For example, patients in Cluster C had higher tumor grade and lower survival rates. Moreover, we visualized the distribution relationship between these clinical characteristics and molecular subtypes using a Sangi diagram (**Figure 3B**), which suggested that molecular subtype can provide novel perspectives to predict progression and outcomes of HCC.

### 3.4 Functional enrichment analysis of molecular subtypes

DEGs for each cluster (Cluster A vs. Cluster B+Cluster C, Cluster B vs. Cluster A+Cluster C, Cluster C vs. Cluster A+Cluster B) were identified and screened according to the criteria of |log2 (Fold Change) |>log2 (1.5) and FDR<0.05. Then, we performed KEGG enrichment analysis through the WebGestalt R package (FDR<0.001). We found that a large proportion of upregulated genes in the three subgroups were enriched in metabolic signaling pathways, such as cholesterol metabolism, tryptophan metabolism and type 1 diabetes mellitus, but that downregulated genes seemed to be more related to immune-related biological activities, such as the T-cell receptor signaling pathway and Th17 cell differentiation (**Supplementary Figures 3A-C**). Notably, the cell cycle, as a significant pathway for both Cluster A and Cluster C, was enriched by downregulated genes in Cluster A, though it was enriched by significantly upregulated genes in Cluster C.

**Figure 4:**
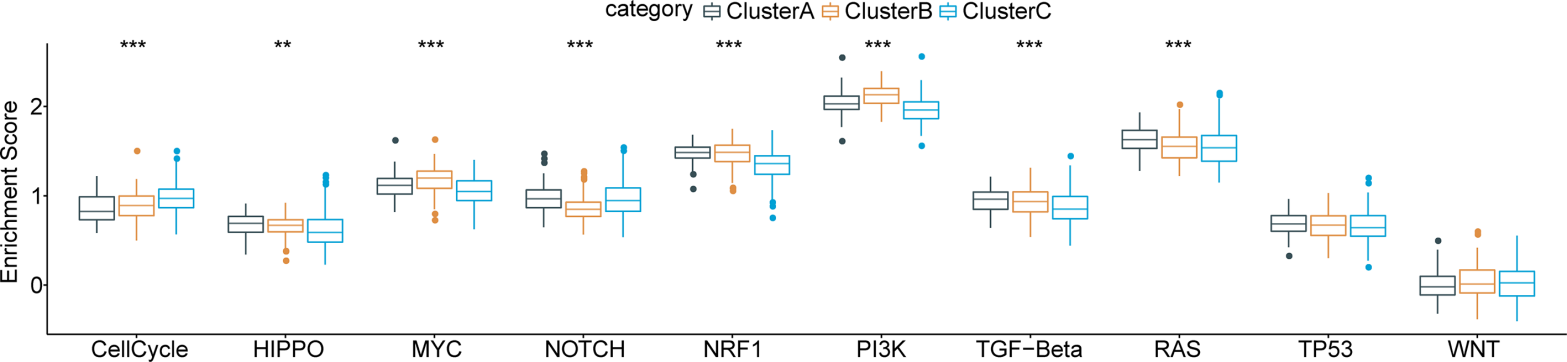
Functional pathway enrichment analysis based on differentially expressed genes in each molecular subtype and 8 of 10 typical tumor-related signaling pathways show significant differences among three subtypes. **p < 0.01; ***p < 0.001.

Furthermore, we obtained characteristic genes of 10 typical tumor-related signaling pathways from a previous study (32) and computed enrichment scores for each patient based on these 10 pathways using the ssGSEA method. Based on the Kruskal test, we detected critical differences in 8 of the 10 oncogenic signaling pathways among the three subtypes (**Figure 4**), indicating a close relationship between molecular subtype and tumor-driving factors.

### 3.5 Comprehensive immune signatures in the three molecular subtypes

**Figure 5:**
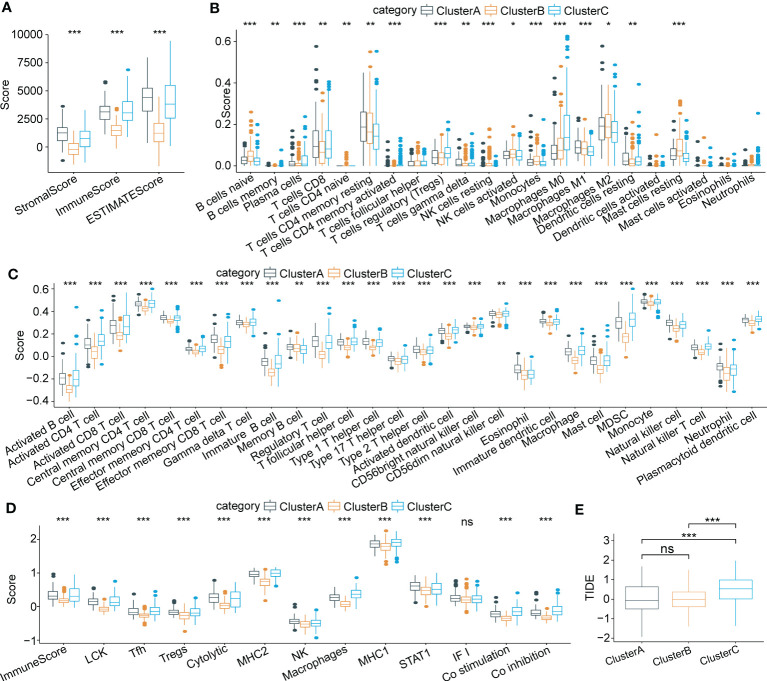
Characteristics of immune cell infiltrating landscape and immunotherapy response in three molecular subtypes. **(A)** Comparison of Stromal score, Immune score and ESTIMATE scores among three subtypes. **(B)** Immune infiltrating characteristics of 22 immune cells among three subtypes. **(C)** and **(D)** Comparison of immune scores based on the characteristic genes of 28 immune cells and 13 immune-associated gene sets among three subtypes. **(E)** Significant differences of TIDE scores among three subtypes. *p < 0.05; **p < 0.01; ***p < 0.001; ns p > 0.05.

To characterize immune signatures in HCC, we calculated the immune infiltration scores of each patient in the LIHC cohort from TCGA. The results showed that patients in Cluster A, with the best prognosis, had the highest immune infiltration levels. Patients in Cluster C had a higher immune score than those in Cluster B (**Figure 5A**), whereas patients in Cluster B had a better prognosis than those in Cluster C. Due to the phenomenon of immune escape in the subtype C, the reason for the worse prognosis of C may not be the lower immune infiltration. Moreover, infiltration of 22 kinds of primary immune cells was evaluated by the CIBERSORT algorithm, showing that the immune infiltration scores of the majority of immune cells among the molecular subtypes differed significantly (**Figure 5B**). For verification, we acquired characteristic genes of 28 immune cells and 13 immune-associated gene sets from previous research (33, 34) and calculated immune scores by ssGSEA. Similarly, significant differences in immune cell infiltration status and immune-related gene sets were identified among the three subtypes (**Figures 5C, D**). Furthermore, we implemented TIDE software to evaluate potential treatment response to immunotherapy. The results showed that Cluster C had a higher prediction score than Cluster B or Cluster A (P < 0.0001) (**Figure 5E**); thus, immune escape was more prone to occur in Cluster C, and it was less possible for patients to benefit from immunotherapy.

### 3.6 Establishment of a risk model based on TAM-related key marker genes

**Figure 6:**
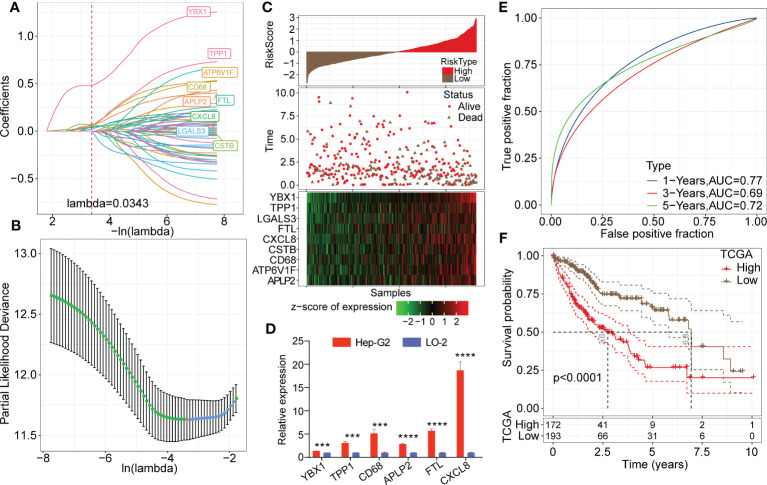
Establishment of the risk model based on key TAM-related genes. **(A)** LASSO coefficients profile plots of each independent variable changing with the lamba value. **(B)** Confidence interval under each lambda value. The vertical axis refers to the partial likelihood deviance and the risk model was optimized as lambda=0.0343. **(C)** Heatmap of Z score expression distribution of 9 genes in the risk model (including TPP1, FTL, CXCL8, CD68, ATP6V1F, CSTB, YBX1, LGALS3, and APLP2). **(D)** Relative transcription levels of YBX1, TPP1, CD68, APLP2, FTL and CXCL8 genes were significantly upregulated in the Hep-G2 cell line compared with the LO-2 cell line (P < 0.05). **(E)** ROC analysis curves of RiskScore in TCGA-LIHC cohort indicate the prognostic classification efficacy of the risk model for 1-, 3- and 5-year survival. **(F)** Kaplan-Meier survival curves of high or low risk groups of HCC patients in TCGA-LIHC cohort.

Univariate Cox analysis of the 165 marker genes of TAMs was performed using the survival R package in TCGA-LIHC cohort, and 58 genes associated with prognosis were identified (P<0.05). We used LASSO regression analysis to compress these 58 key genes. The changing trajectory of each argument is displayed in **Figure 6A**, from which we found that as the lambda value increased, the number of argument coefficients approaching 0 gradually increased. The confidence intervals for each lambda value are shown in **Figure 6B**, and we found that the risk model was optimized as lambda=0.0343. Finally, 9 genes were identified as target genes: TPP1, FTL, CXCL8, CD68, ATP6V1F, CSTB, YBX1, LGALS3, and APLP2. We calculated the coefficients of these 9 genes through multivariate Cox regression analysis and determined the final calculation formula as follows: RiskScore = 0.108*TPP1 + 0.133*FTL + 0.059*CXCL8 + 0.072*CD68 + 0.108*ATP6V1F + 0.072*CSTB + 0.488*YBX1 + 0.055*LGALS3 + 0.166*APLP2.

### 3.7 Predictive efficiency evaluation of the risk model in training datasets

Then, we calculated the risk score of each patient in the LIHC cohort from TCGA. As shown in **Figure 6C**, patients with high RiskScore had an obviously lower survival rate than those with low RiskScore. Z score analysis was also conducted, and samples with scores greater than zero were classified into the high-risk group; the other samples were classified into the low-risk group. A heatmap showed that the Z score of the expression of 9 genes correlated positively with poor prognosis. Moreover, we measured relative transcription levels of 9 genes in hepatoma cell lines (Hep-G2 and Huh-7) and normal hepatic cell line by RT−qPCR. The results showed that the YBX1, TPP1, CD68, APLP2, FTL and CXCL8 genes were significantly upregulated in the Hep-G2 cell line compared with the LO-2 cell line (P < 0.05) (**Figure 6D**). Additionally, the relative transcription levels of CSTB, CD68, FTL, LGALS3, TPP1 and APLP2 genes were found to be upregulated in the Huh-7 cell line compared with the LO-2 cell line (**Supplementary Figure 4**).

**Figure 7:**
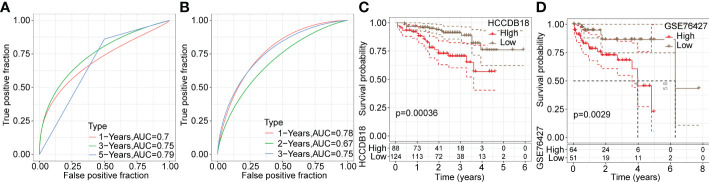
Validation of the reliability of risk model in training datasets. **(A, B)** ROC analysis curves of the risk model in the LIHC cohort from HCCDB and the independent GSE76427 dataset. **(C, D)** Kaplan-Meier survival curves of the risk model in the LIHC cohort from HCCDB and the independent GSE76427 dataset.

Next, ROC analysis was performed to evaluate the prognostic classification efficacy of the risk model for 1-, 3- and 5-year survival. As shown in **Figure 6E**, the risk model had high areas under the positive fraction curve, indicating excellent predictive performance. Moreover, we plotted a Kaplan−Meier (KM) survival curve, which showed a significant difference in prognosis between the high- and low-risk groups (**Figure 6F**). In validation of the reliability of the risk model, we obtained similar results after applying the 9-gene risk model for patients in the LIHC cohort from HCCDB and independent dataset GSE76427 (**Figures 7A–D**), suggesting that the risk model is a significant factor for predicting prognosis.

### 3.8 Indicative value of the risk model for clinical phenotype in HCC

**Figure 8:**
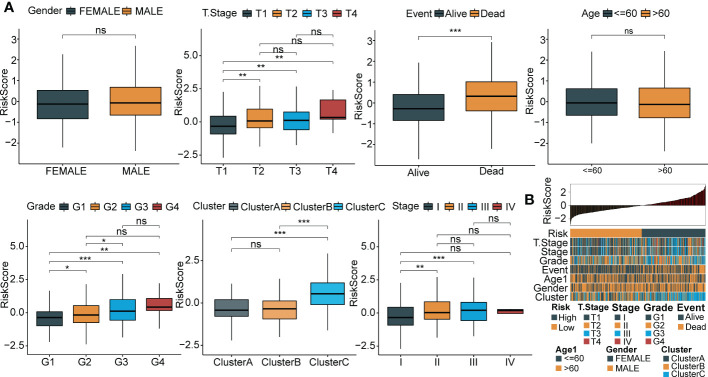
Relationship between RiskScore and clinical phenotypes of HCC patients. **(A)** Differences of RiskScore between multiple clinicopathologic groups in the TCGA-LIHC cohort (gender, stage, survival, age, grade, cluster and grade). **(B)** The distribution map of phenotypic characteristics of the samples with an increase of RiskScore. *p < 0.05; **p < 0.01; ***p < 0.001; ns p > 0.05.

To explore the association between RiskScore and clinicopathological phenotypes of patients with HCC, we analyzed differences in RiskScore among diverse clinical phenotypes in the LIHC cohort from TCGA. The results showed that an increased RiskScore had a critically positive correlation with advanced tumor stage, increased patient mortality and high tumor grade (**Figure 8A**). Moreover, Cluster C had the highest RiskScore among the three molecular subtypes. **Figure 8B** depicts the distribution map of different phenotypic characteristics with an increased RiskScore.

### 3.9 Functional enrichment analysis and immune signature of the risk model in HCC

**Figure 9:**
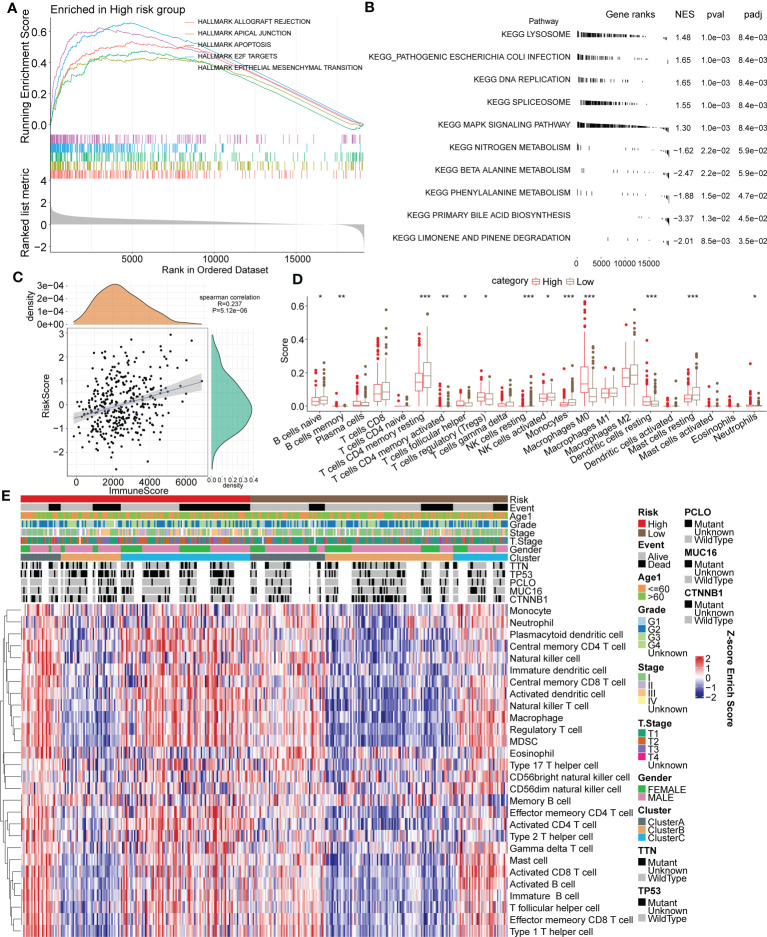
Association between functional signaling pathways and the immune signature and RiskScore in HCC. **(A)** The five most significantly enriched pathways of GSEA enrichment analysis in high or low risk group based on HALLMARK gene sets. **(B)** The five most significantly enriched pathways of KEGG enrichment analysis in high or low risk group. **(C)** Correlation analysis of RiskScore and the ESTIMATE immune score. **(D)** Immune infiltrating scores of 22 immune cells in high- and low-risk groups. **(E)** Heatmap for the 28 primary immune cell enrichment scores in groups of different taxonomic characteristics. *p < 0.05; **p < 0.01; ***p < 0.001.

Gene sets related to HALLMARK and KEGG were downloaded from the publicly available GSEA website. First, we conducted GSEA gene enrichment analysis on the HALLMARK dataset through the ClusterProfiler R package. In the high-risk group, 37 functional pathways were significantly enriched; conversely, none were enriched in the low-risk group. We selected the first five most significant pathways for visualization (**Figure 9A**). More importantly, enriched pathways such as apoptosis and the epithelial-mesenchymal transition (EMT) play essential roles in the oncogenesis and progression of tumors. The first five most significant pathways of the KEGG enrichment analysis for both groups are displayed in **Figure 9B**.

The ESTIMATE algorithm and Spearman analysis were conducted to evaluate the correlation between tumor immune status and the risk model. The results showed a positive correlation between RiskScore and the immune ESTIMATE score (R value=0.237. P value < 0.001) (**Figure 9C**). Moreover, the immune scores of 22 primary immune cells were computed by the CIBERSORT algorithm, and the infiltrating levels of most immune cells differed between the high- and low-risk groups (**Figure 9D**). Notably, the immune scores of B cells, CD4+ T cells and NK cells in the high-risk group were significantly lower than those in the low-risk group, but they were higher for Tregs. The distribution of 28 primary immune cell enrichment scores in groups of different taxonomic characteristics is shown in a heatmap in **Figure 9E**. Importantly, there were more samples of the Cluster B subtype distributed in the low-risk group, with lower TIDE scores, and there were more samples of the Cluster C subtype distributed in the high-risk group, with higher TIDE scores. In a previous analysis, we elucidated that tumor immune escape is more prone to occur in Cluster C. The above results suggested the favorable value of the risk model for tumor immune assessment.

### 3.10 RiskScore combined with clinical phenotype improves the predictive efficacy of the prognostic model in HCC

**Figure 10:**
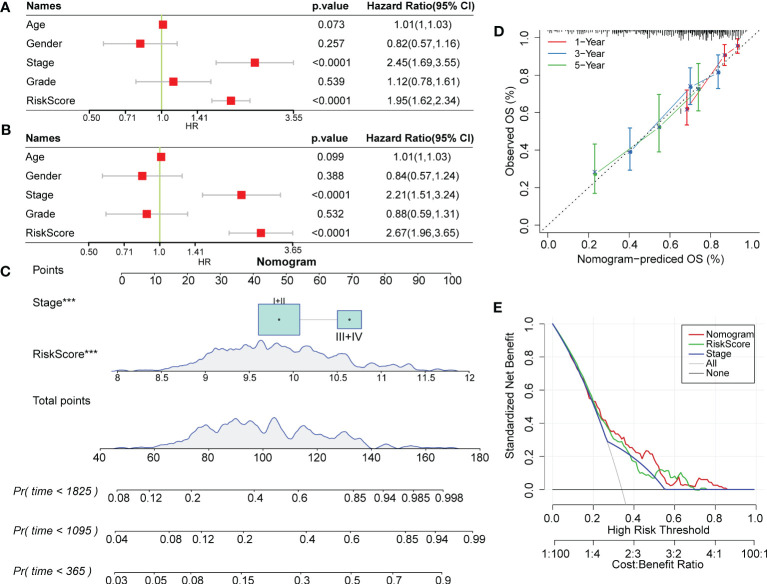
RiskScore combined with clinical phenotype improved the predictive efficacy of the prognostic model in HCC. **(A)** and **(B)** Univariate and multivariate Cox regression analysis of the risk score and the clinical phenotype was determined by the P value and hazard ratio. **(C)** Nomogram model established by combining with RiskScore and other clinicopathological features. **(D)** The predicted calibration curve approached the standard curve at the 1-, 3- and 5-year calibration points. **(E)** Decision curve of the nomogram.

RiskScore was proven to be a significantly independent prognostic factor through Cox regression analysis with multiple clinical features (**Figures 10A, B**). To quantify prognostic evaluation and survival prediction of patients with HCC, we combined it with other clinicopathological traits to construct a nomogram (**Figure 10C**). From the constructed model, RiskScore was the greatest predictor for survival in patients. Furthermore, a calibration curve was generated to assess the predictive performance of the model. As illustrated in **Figure 10D**, the predicted calibration curve approached the standard curve at three calibrated points (1, 3 and 5 years), indicating that the nomograph had excellent predictive ability. Furthermore, a decision curve was utilized to validate the stability of the model. The results showed that the predictive benefits of the risk score and nomogram were critically superior to those of the extreme curve (**Figure 10E**), displaying the strongest survival prediction ability.

## 4 Discussion

HCC is one of the most refractory malignancies worldwide, with complex and multiple risk factors involved in its pathogenesis (35, 36). Currently, the main treatment for patients with HCC is surgical hepatectomy and liver transplantation. Unfortunately, most patients are at an advanced stage when diagnosed, and only 5% to 15% of patients are eligible for surgical resection (37). Owing to drug resistance and chemotherapy toxicity, a minority of patients can benefit from chemotherapy (37). The lack of effective and safe treatment for patients at advanced stages leads to rapid disease progression and poor prognostic outcomes (11). It has been widely evidenced that the immune system exerts essential functions in antitumor processes, and improved insights into tumor immunobiology have brought about novel treatment options for patients. TIME-regulated immunotherapy has a variety of clinical advantages, including triggering a systemic, effective and lasting antitumor immune response with a low recurrence rate and few side effects (38, 39). As a highly immunogenic malignancy, HCC is characterized by abundant immune cell infiltration into the tumor microenvironment. Various immunotherapeutic strategies, including adoptive cell therapy, tumor vaccines and immune checkpoint inhibitors, have achieved certain success in HCC (9, 40, 41).

As important components in the TIME, macrophages have been proven to directly or indirectly regulate key characteristics of malignancies, including angiogenesis, metastasis, formation of the tumor microenvironment and drug resistance. A previous study demonstrated that IL-6 generated by TAMs induces upregulation of CD47 on hepatoma cells *via* the STAT3 signaling pathway, subsequently influencing TAM-mediated phagocytosis, promoting tumor progression in HCC, and leading to poor prognosis (42). Zong et al. (43) found that M1 TAMs exerts oncogenic effects by enhancing expression of programmed cell death ligand (PD-L) 1, a pivotal immune checkpoint molecule mediating HCC immune escape. In our study, we performed cluster dimensionality reduction to identify different cell subpopulations through specifically expressed marker genes; we then systematically explored the relationship between TAM-related marker genes and clinical phenotype, survival prognosis and TIME in patients with HCC. We determined three molecular subtypes by calculating the consistency matrix and consistency CDF. We found that the prognosis of patients in Cluster A was much better than that of patients in Cluster B or Cluster C in both the LIHC cohort from TCGA and independent dataset GSE76427, with a higher immune score, which was consistent with previous research (33, 34). The immunosuppressive microenvironment and tumor immune escape might be enhanced in Cluster C. In addition, we obtained characteristic genes of 10 tumor-related signaling pathways from a previous study (32) and found critical differences in 8 of the 10 oncogenic signaling pathways among the three subtypes, indicating that molecular subtype has good predictive efficacy with regard to molecular functions and biological activities.

Single-cell sequencing technology has been emerging as an innovation in biomedical research and clinical practice, enabling comprehensive characterization of cell subpopulation, status and lineage in heterogeneous tissues (18, 44). Indeed, identification of cell subpopulations modulating phenotype is essential for the study of disease progression, tumor metastasis, therapeutic response and survival probability evaluation (45, 46). Therefore, single-cell sequencing can greatly promote the discovery of targeted therapy and prognostic biomarkers. However, analyzing vast amounts of scRNA-seq data is notably challenging work for investigators; hence, it is necessary to implement systems biology approaches through mathematical models (47, 48). In this work, we performed unsupervised cluster analysis to identify distinct cell subtypes and, more importantly, established a quantitative and stable risk model based on the GEO-LIHC scRNA-seq dataset for predicting survival probability. Surprisingly, the risk model combined with clinicopathological features showed better prediction performance for the 1-, 3- and 5-year survival of patients with HCC. Among the target genes identified by LASSO Cox regression analysis, CSTB was specifically upregulated in HCC, and levels of CSTB and alpha-fetoprotein (AFP) may serve as a highly sensitive diagnostic biomarker for HCC (49) patients. Similarly, overexpressed YBX1 was found to be a master oncogenic contributor correlating highly with tumor progression and prognostic outcomes (50, 51). Moreover, Zhang et al. (52) demonstrated that LGALS3 secreted by HCC cells facilitates the metastatic property of hepatoma cells and reduces the bone metastasis-free survival of patients.

In addition to predicting the prognostic outcomes of patients, the risk signature showed an association with the immune landscape and immunotherapeutic response in HCC. We calculated scores of immune infiltrating cells through CIBERSORT and ESTIMATE. Notably, the immune scores of B cells, CD4+ T cells and NK cells in the high-risk group were lower than those in the low-risk group, though Tregs were present at higher levels in the high-risk group. Significant advances have been made in understanding the essential roles of NK cells in HCC. By killing cancer cells or enhancing the adaptive T-cell immunological response, NK cells exert powerful antitumor effects in early stages (53–55). Tregs have been widely reported to be a tumor immunosuppressive factor, and depletion of Tregs is an attractive strategy for HCC (56). This might explain why the infiltrating level of Tregs was higher in high-risk score patients with HCC. Intriguingly, we found more samples of the Cluster C subtype in the high-risk group, indicating that patients with high RiskScore might benefit less from immunotherapy than those with low RiskScore. According to the prognostic results, the immune score in Cluster C seems to be lower. However, due to the phenomenon of immune escape in the Cluster C, the reason for the worse prognosis of patients may not be the lower immune infiltration. This also indirectly indicates that in high-risk group, immune escape may be more prone to occur, which mainly related to Cluster C. The differential distribution of multiple immune cells might provide new perspectives on immunotherapy for HCC.

Collectively, this study aimed to cluster patients with HCC into different TAM-related subtypes and establish a risk model to link TAM-related marker genes with prognosis, immune characteristics and biological activities. Through multidimensional and multidataset validation, the novel signature we identified showed excellent prospects for predicting the prognosis of patients with HCC. However, our study has some limitations. First, the sample size was fairly small, and the stability and accuracy of the risk model need to be verified in a larger sample with multiple space-time distributions. Second, the data analyzed were generated from tumor tissues of patients with HCC, with low diagnostic efficacy for early HCC. To improve clinical applicability, the predictive efficiency of the risk model for peripheral circulating immune cells in HCC patients needs to be evaluated. In future research, we will explore the functional roles and underlying mechanisms of these 9 key genes in HCC through phenotypic assays and molecular biology experiments (both *in vivo* and *in vitro*).

## 5 Conclusions

In conclusion, we constructed a novel gene signature based on TAM-related marker genes, which was validated to be stable and highly efficient for predicting prognostic outcomes, immune signature and biological activities in HCC. Our study suggests effective strategies for immunotherapeutic therapy and prognostic intervention.

## Data availability statement

The original contributions presented in the study are included in the article/**Supplementary Material**. Further inquiries can be directed to the corresponding author.

## Author contributions

LL designed and guided the study. YSS, CX, and XG wrote and edited the manuscript. YSS and CX conducted data analysis and plotted graphics. WW, YS, and RZ helped with reference collection. All authors contributed to the article and approved the submitted version.

## Supplementary material

Supplementary Figure 1 Infiltration and cluster dimension reduction of scRNA-seq data. **(A)** Bar chart of cell count statistics before and after filtration. **(B)** The anchor plots of the top 50 PCs for PCA dimensionality reduction. **(C)** UMAP distribution map of all samples before excluding batches. **(D)** UMAP distribution map of all samples after excluding batches.

Supplementary Figure 2 Violin map of marker gene expression of 16 cell subgroups.

Supplementary Figure 3 Gene enrichment analysis based on differentially expressed genes. **(A-C)** Bubble diagrams of KEGG enrichment analysis for upregulated and downregulated genes in the three molecular subtypes.

Supplementary Figure 4 Relative transcription levels of CSTB, CD68, FTL, LGALS3, TPP1 and APLP2 genes were upregulated in the Huh-7 cell line compared with the LO-2 cell line.
