# Supplementary figures and images for: Identification of a novel signature based on macrophage-related marker genes to predict prognosis and immunotherapeutic effects in hepatocellular carcinoma
Source: Front Oncol. 2023 May 25;13:1176572. doi: 10.3389/fonc.2023.1176572 (PMC10248258; doi:10.3389/fonc.2023.1176572)

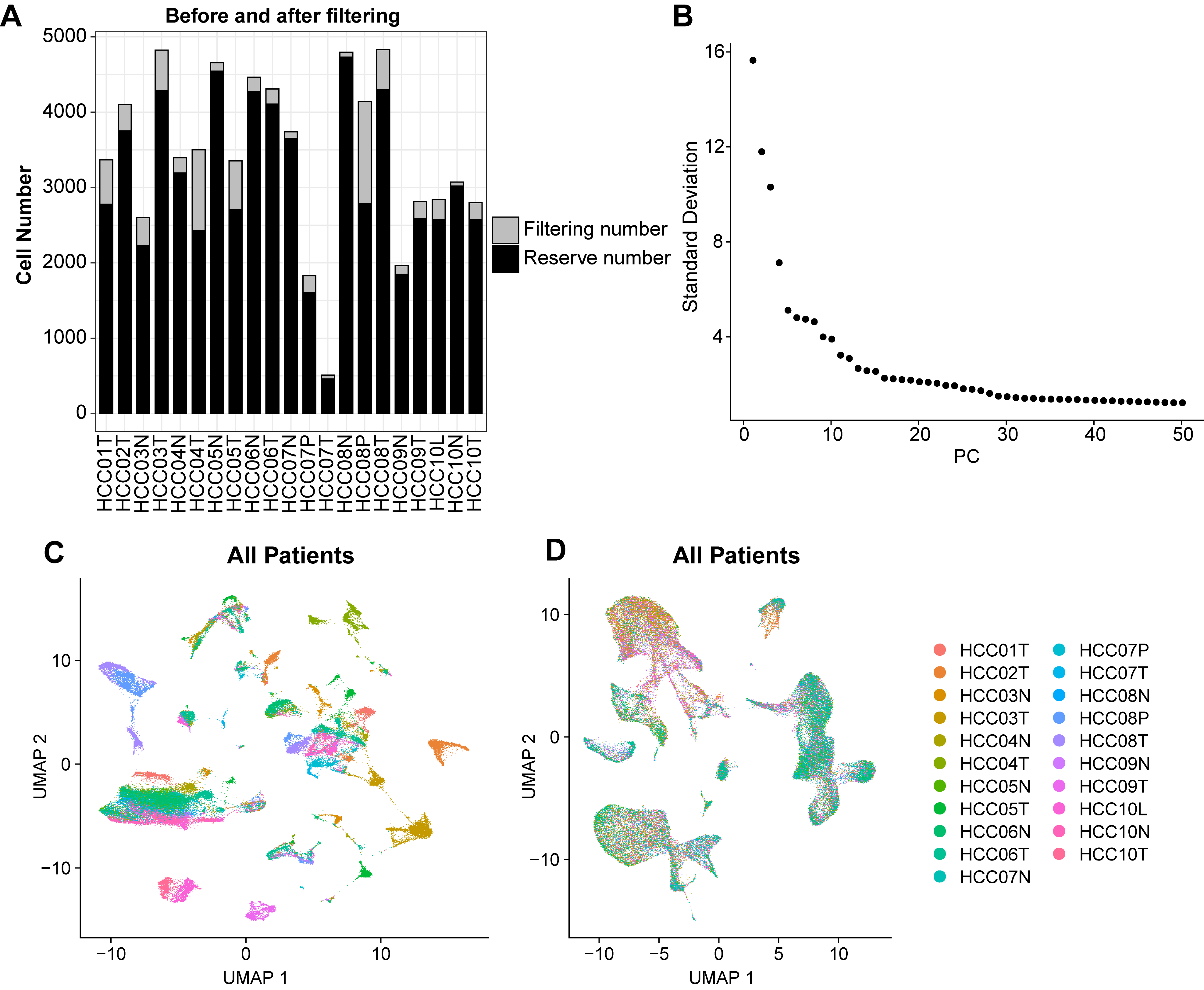

Supplement: Supplementary Figure 1 — Infiltration and cluster dimension reduction of scRNA-seq data. (A) Bar chart of cell count statistics before and after filtration. (B) The anchor plots of the top 50 PCs for PCA dimensionality reduction. (C) UMAP distribution map of all samples before excluding batches. (D) UMAP distribution map of all samples after excluding batches. [file Image_1.tif]

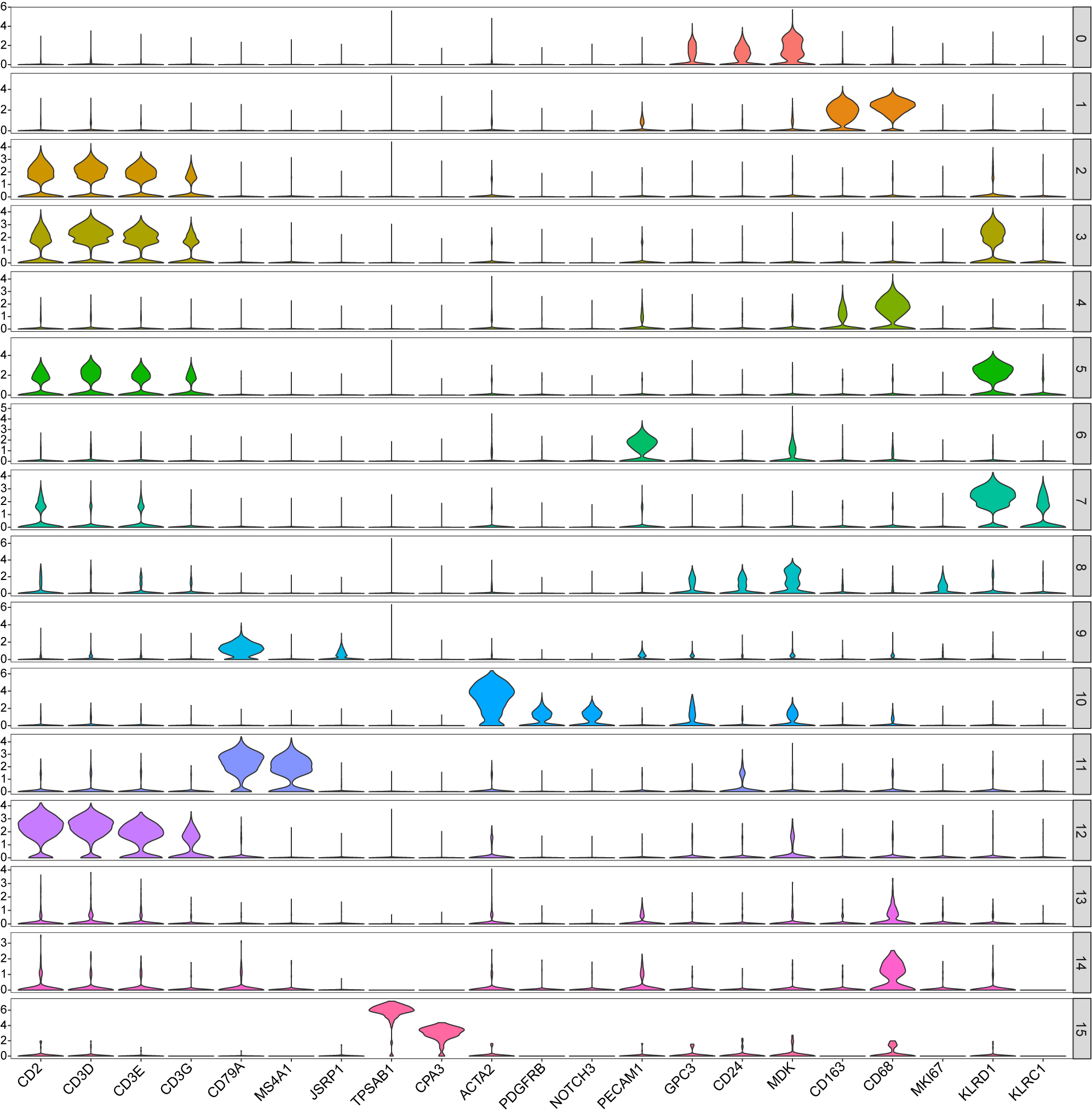

Supplement: Supplementary Figure 2 — Violin map of marker gene expression of 16 cell subgroups. [file Image_2.tif]

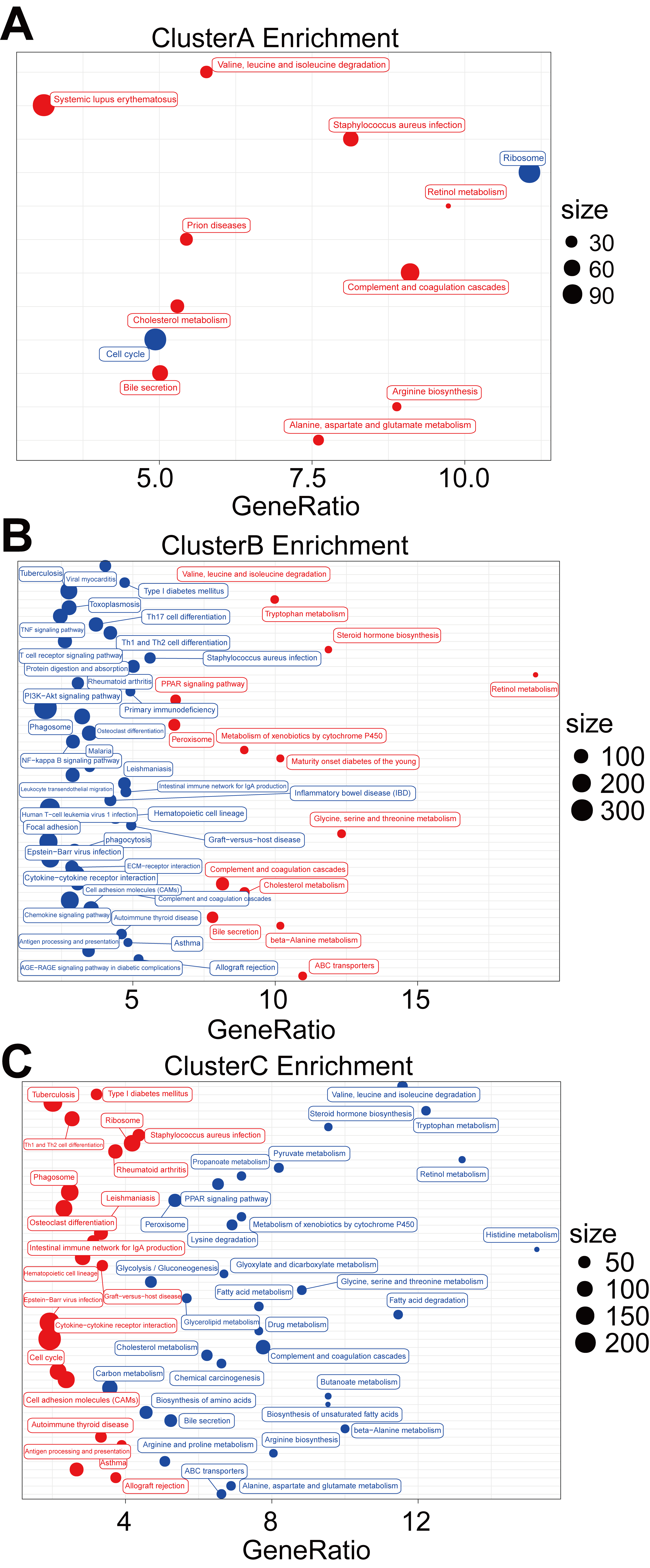

Supplement: Supplementary Figure 3 — Gene enrichment analysis based on differentially expressed genes. (A-C) Bubble diagrams of KEGG enrichment analysis for upregulated and downregulated genes in the three molecular subtypes. [file Image_3.tif]

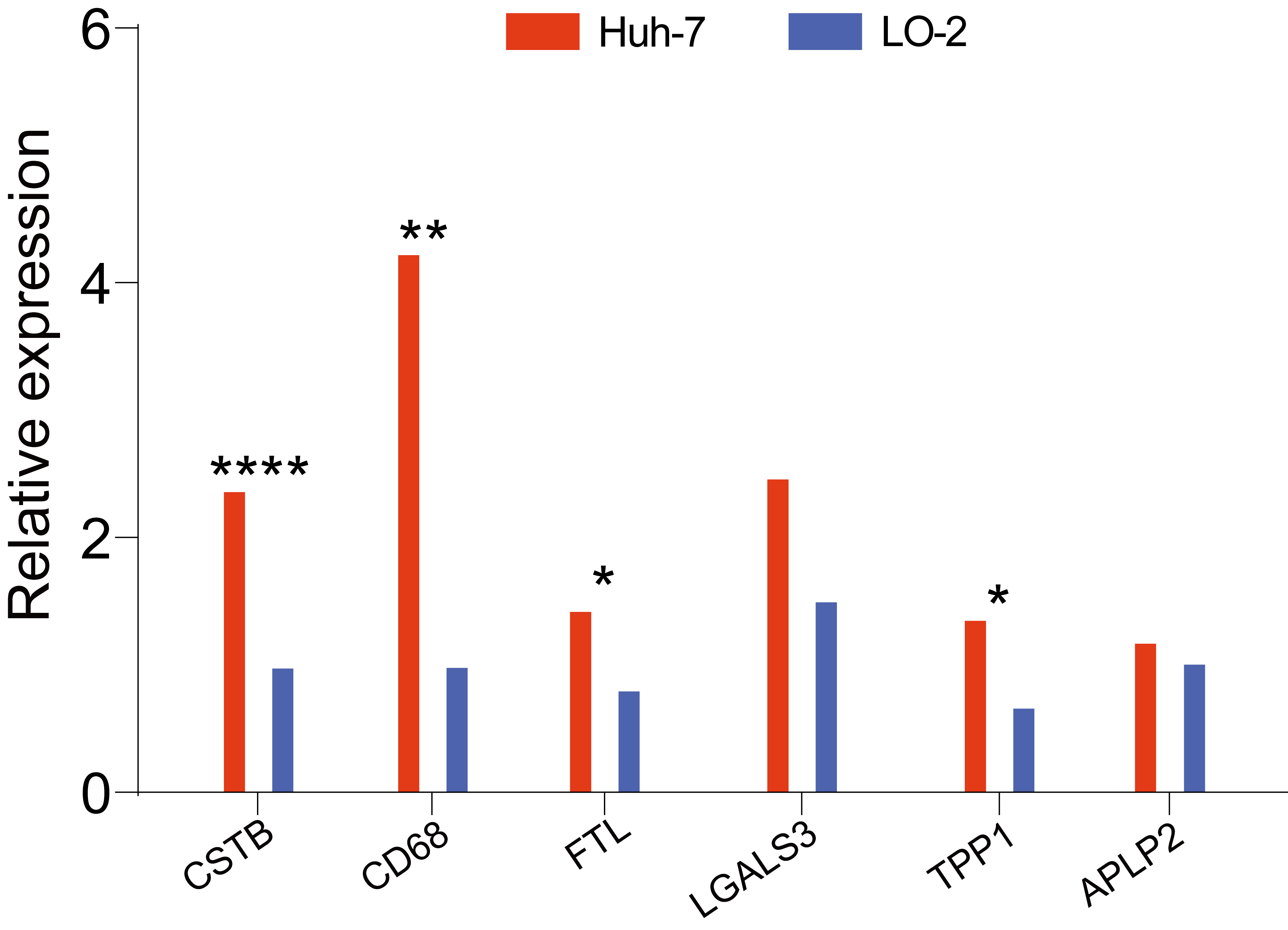

Supplement: Supplementary Figure 4 — Relative transcription levels of CSTB, CD68, FTL, LGALS3, TPP1 and APLP2 genes were upregulated in the Huh-7 cell line compared with the LO-2 cell line. [file Image_4.tif]
