# Supplementary material for: Identification of a novel signature based on macrophage-related marker genes to predict prognosis and immunotherapeutic effects in hepatocellular carcinoma
Source: Front Oncol. 2023 May 25;13:1176572. doi: 10.3389/fonc.2023.1176572 (PMC10248258; doi:10.3389/fonc.2023.1176572)
Supplement: Supplementary file 5 [file Table_1.docx]

Supplementary Table 1. The information of primer sequences for RT-qPCR assay.

| Primer name | Sequence (5'-3') |
| --- | --- |
| GAPDH-F | GGAGCGAGATCCCTCCAAAAT |
| GAPDH-R | GGCTGTTGTCATACTTCTCATGG |
| YBX1-F | GAAGTGATGGAGGGTGCTGACAAC |
| YBX1 -R | GGTTTAGGGTTTTCTGGGCGTCTG |
| TPP1-F | GGACTTTCTGACTTGCTGGCTGAG |
| TPP1-R | AGGCTTGGCTGTTATTGCTGGTG |
| CD68-F | GCTACATGGCGGTGGAGTACAATG |
| CD68-R  APLP2-F  APLP2-R  FTL-F  FTL-R  CXCL8 –F  CXCL8 –R  LGALS3-F  LGALS3-R  CSTB –F  CSTB –R  ATP6V1F-F  ATP6V1F-R | CGATGATGAGAGGCAGCAAGATGG  GCTGTGGATGAGGATGATGAGGATG  GTTGGCAGAGGAGTTGGAGGAATC  GCGATGATGTGGCTCTGGAAGG  TGTGGAGGTTGGTCAGGTGGTC  CTCTCTTGGCAGCCTTCCTGATTTC  CAGTTTTCCTTGGGGTCCAGACAG  AGCACCTGCACCTGGAGTCTAC  TGAAGCGTGGGTTAAAGTGGAAGG  AGGTGAGGTCCCAGCTTGAAGAG  CATCATGCTTGGCTTTGTTGGTCTG  CTCATCGCAGTGATCGGAGAC  CGGTTCTTGTTAAGCTCCCCTA |
